# Tweeting about public health policy: Social media response to the UK Government’s announcement of a Parliamentary vote on draft standardised packaging regulations

**DOI:** 10.1371/journal.pone.0211758

**Published:** 2019-02-26

**Authors:** Jenny L. Hatchard, Joao Quariguasi Frota Neto, Christos Vasilakis, Karen A. Evans-Reeves

**Affiliations:** 1 Tobacco Control Research Group, Department for Health, University of Bath, Bath, United Kingdom; 2 AMBS, University of Manchester, Manchester, United Kingdom; 3 Centre for Healthcare Innovation and Improvement (CHI2), School of Management, University of Bath, Bath, United Kingdom; The University of Hong Kong, HONG KONG

## Abstract

**Background:**

Standardised tobacco packaging has been, and remains, a contentious policy globally, attracting corporate, public health, political, media and popular attention. In January 2015, the UK Government announced it would vote on draft regulations for the policy before the May 2015 General Election. We explored reactions to the announcement on Twitter, in comparison with an earlier period of little UK Government activity on standardised packaging.

**Methods:**

We obtained a random sample of 1038 tweets in two 4-week periods, before and after the UK Government’s announcement. Content analysis was used to examine the following Tweet characteristics: support for the policy, purpose, Twitter-user’s geographical location and affiliation, and evidence citation and quality. Chi-squared analyses were used to compare Tweet characteristics between the two periods.

**Results:**

Overall, significantly more sampled Tweets were in favour of the policy (49%) in comparison to those opposed (19%). Yet, at Time 2, following the announcement, a greater proportion of sampled tweets opposed standardised packaging compared to the period sampled at Time 1, prior to the announcement (p<0.001). The quality of evidence and research cited in URLs linked at Time 2 was significantly lower than at Time 1 (p<0.001), with peer-reviewed research more likely to be shared in positive Tweets (p<0.001) and in Tweets linking to URLs originating from the health sector (p<0.001). The decline in the proportion of positive Tweets was mirrored by a reduction in Tweets by health sector Twitter-users at Time 2 (p<0.001).

**Conclusions:**

Microblogging sites can reflect offline policy debates and are used differently by policy proponents and opponents dependent on the policy context. Twitter-users opposed to standardised packaging increased their activity following the Government’s announcement, while those in support broadly maintained their rate of Twitter engagement. The findings offer insight into the public health community’s options for using Twitter to influence policy and disseminate research. In particular, proliferation of Twitter activity following pro-public health policy announcements could be considered to ensure pro-health messages are not overshadowed by anti-regulation voices.

## Introduction

Twitter is a global social media microblogging tool allowing millions of users to share short online posts instantly. User numbers have grown rapidly from 140 million users in 2012 [1] to 326 million monthly active users in 2018[2]. Vast amounts of data are generated, which are free and accessible for non-commercial purposes, and therefore appealing for social, political, cultural and economic research [1, 3–5]. On health research, the potential of Twitter data to support public health initiatives has been explored[6] and Twitter has been used *inter alia* to examine the spread of diseases[7], childhood obesity [8], e-cigarettes[9, 10] and diabetes[11].

Standardised tobacco packaging (Box 1) has attracted popular, political and corporate interest wherever it has been considered[12–15]. In the UK, Government consultations in 2012 and 2014 prompted supporters and opponents of standardised packaging to submit lengthy consultation responses and undertake extensive lobbying and communications campaigns[16–19].

**Box 1**

Standardised packaging entails the mandatory removal of brand images, colours and messages from tobacco product packs. Instead, packs are required to be the same size, shape, style and colour (drab brown or green), with all brand names and variants printed in a prescribed typeface and font size[20, 21] and include text and pictorial health warnings. As of July 2018, Australia, the UK, France, Ireland, Norway, New Zealand and Hungary had all implemented the policy and Slovenia had legislated with implementation planned for 2020; Brazil, Canada, Chile, Ecuador, Georgia, Panama, Romania, Thailand and Uruguay were all progressing towards legislation, and many more countries were considering the policy [22, 23].

The political debate hinged on the evidence base for standardised packaging. Independent evidence reviews commissioned by the UK and Irish Governments concluded that the measure was highly likely to deter youth smoking uptake[24–26]. Evidence from Australia following implementation showed that standardised packaging reduces pack display and appeal[27, 28], increases quit attempts and health warning effectiveness[29–31], helps correct misperceptions of harm[28], and (contrary to tobacco industry arguments) does not increase illicit tobacco purchases[32]. However, in the UK, transnational tobacco companies (TTCs) sought to misrepresent the evidence for standardised packaging and to move political attention towards an alternative, lower quality evidence base, which they claimed supported their arguments that standardised packaging would not work and would have ‘negative unintended consequences’ for the economy and illicit trade[33–35]. These are similar arguments to those the industry have used against other tobacco control policies[36–38].

**Fig 1:**
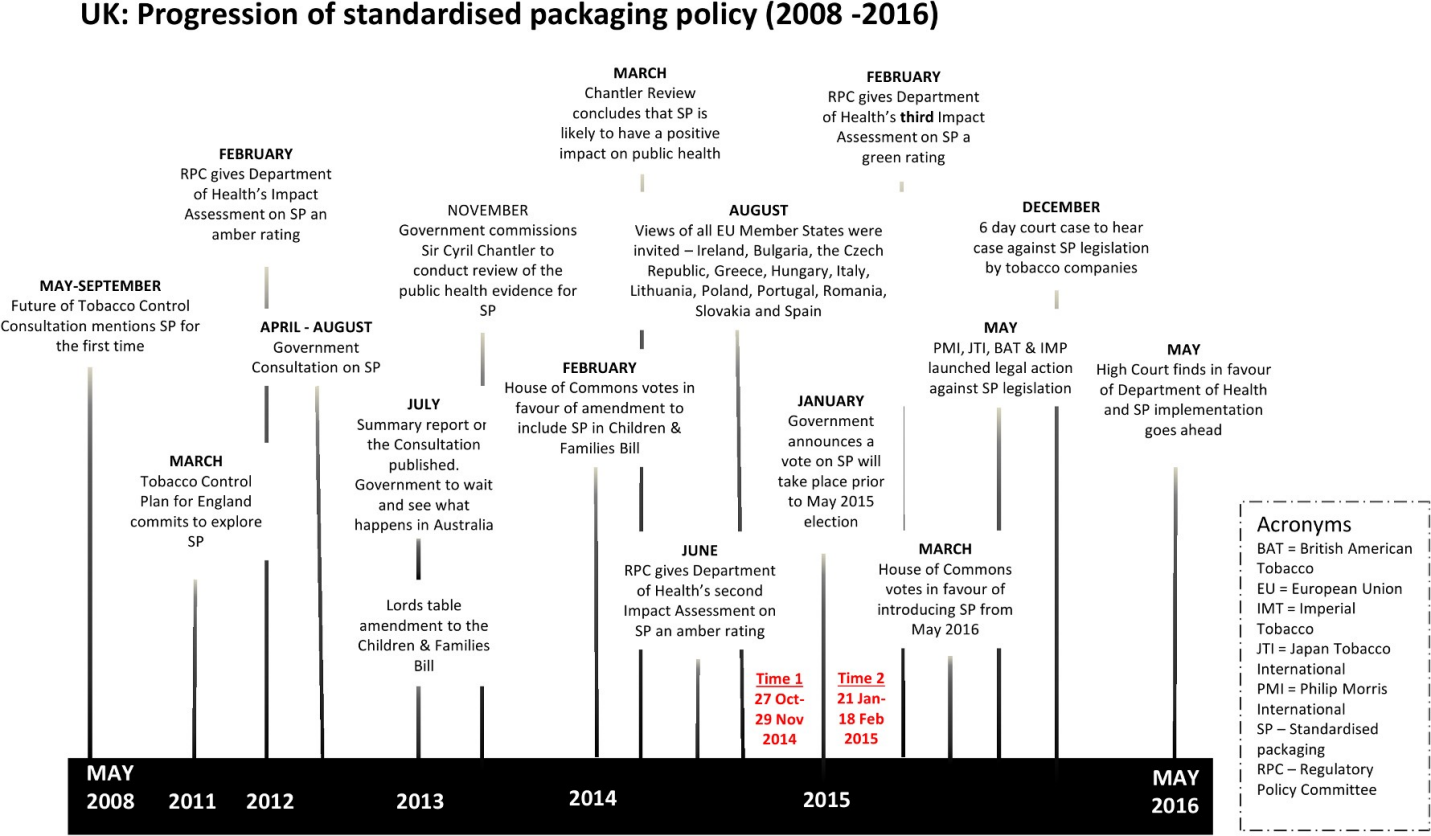
UK progression of standardised packaging policy (2008–2016) and the two time periods data were collected (Time 1 and 2).

The UK Government kept up to date with these evidence debates by undertaking regulatory impact assessments, keeping a watching brief on the impact of the policy in Australia and citing ‘the evidence’ in a series of interim policy decisions (Fig 1). In July 2013 an unexpected decision was made to ‘wait and see’ what evidence emerged from Australia[39]. Then, in March 2014 the Government-commissioned independent ‘Chantler Review’ of the evidence on standardised packaging was published, which ultimately supported standardised packaging[24]. Finally, on the 21st January 2015, the controversy over evidence was provisionally settled by the government’s decision to ‘back the public health case for introducing the policy’. “Having considered all the evidence, the Secretary of State and I believe that the policy is a proportionate and justified response to the considerable public health harm from smoking tobacco” (UK Public Health Minister, Jane Ellison MP)[40]. However, the accompanying announcement to hold a vote in Parliament on standardised packaging before the general election scheduled for May 2015, prompted renewed debate[41].

So far, despite the volume and vehemence of both opposition to and support for the policy, no research has been conducted on whether proponents or opponents of standardised packaging used social media as a campaign tool or simply to voice their opinions. The present study aimed to explore global Twitter communication relating to public health policy change, by examining the case study of standardised tobacco packaging policy in the UK (Box 1). The study examined whether and how the volume, sentiment and purpose of Tweets about standardised packaging of tobacco changed following the announcement of a parliamentary vote on the policy (Fig 1)[20, 40]. Responding to debates relating to the evidence base for standardised packaging[33–35], the study also examined the presence and quality of evidence and research disseminated on Twitter before and after the announcement to explore any differences between proponents and opponents of the policy during a key policy event which could have implications for future tobacco control activities.

## Methods

Quantitative content analysis was used to explore how views on standardised packaging were expressed and shared on Twitter; particularly whether Tweet characteristics changed after the Government’s policy announcement. Ethical approval was obtained from the University of Bath's Department for Health Research Ethics Committee.

### Data collection

Data were collected using Twitter’s Application Programming Interface (API) and the search terms “plain”, “generic”, “standardized”, “standardised”, “standard” AND “pack*”, “tobacco”, “consultation”, “smok*”, “cig*”, “fags” in all combinations and variants. No search restrictions were placed on geographical location of Tweets. Twitter’s terms of service were complied with. Data were streamed using a script developed in R statistical package[42].

### Data sampling and coding

**Fig 2:**
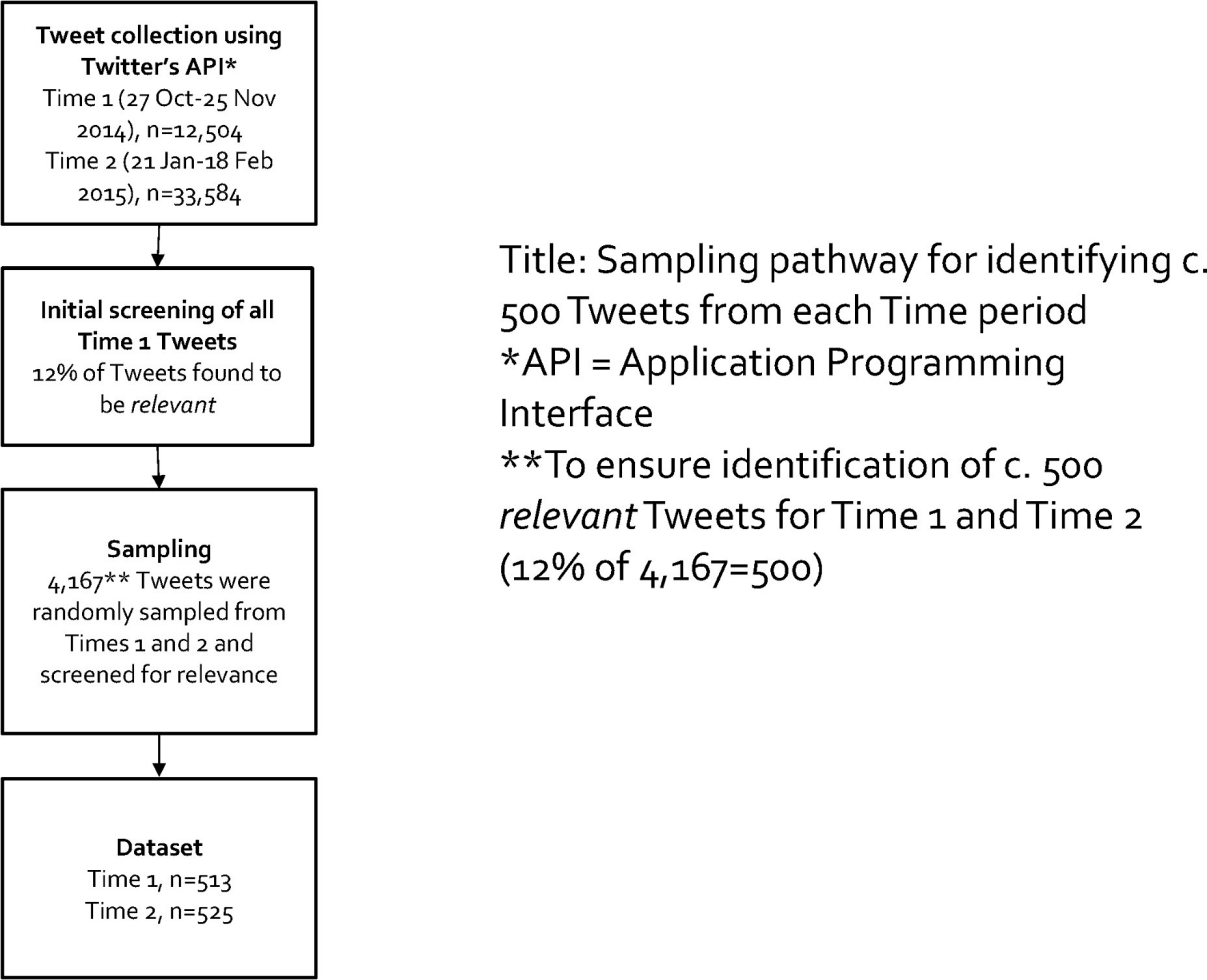
Sampling pathway for identifying c. 500 Tweets from each Time period.

Tweets were collected in two four-week periods, 27 October to 25 November 2014 (Time 1: n = 12,504 tweets) and 21 January to 18 February 2015 (Time 2: n = 33,584 tweets) (Fig 2). At Time 1, the UK had completed its consultation on the regulations[17] and submitted them to the European Union for approval (the 2015/1535 procedure). During this period there were no UK Government announcements on standardised packaging. Time 2 began with the UK Government’s announcement that there would be a Parliamentary vote on standardised packaging prior to the May 2015 General Election[40]. This prompted a period of frequent press coverage and online comment [43].

We aimed to code a sample of 500 Tweets for each of Times 1 and 2. All Time 1 Tweets (n = 12,504) gathered by the API programme using the aforementioned search terms were manually screened for relevance to standardised packaging of tobacco products. All Tweets were read by two coders (CV and JQFN) and coded as relevant, not relevant, unsure. Tweets coded as unsure were reviewed and inclusion or exclusion agreed by the whole team. Of all screened Tweets, 12% were found to be relevant. In order to capture approximately 500 Tweets for each of Times 1 and 2 (taking the 12% accuracy of the algorithm into account), we took a random sample of 4,167 Tweets from each time period (12% of 4,167 = 500).

Screening for relevance within the random samples identified 513 Tweets from Time 1 and 525 from Time 2. At 19% of the estimated total relevant Tweets, this was a sufficiently large sample for the study given that smaller Twitter sample sizes of 0.95% and 9.6% have been found to be suitable for event detection, sentiment analysis and Tweet summarization[44]. The dataset was reviewed for indicators of the presence of social bots[45, 46], but no compelling evidence of automated Tweeting was observed. Both Tweets and Retweets were included in the dataset.

All relevant Tweets were fine-coded for sentiment towards standardised tobacco packaging, purpose of Tweet, Twitter-user geographical location and affiliation (taken from metadata accompanying Tweets), mention of evidence and presence of link to a URL (Table 1). Linked URL webpages and images were coded for quality of evidence cited and author sector. Tweets were coded part deductively informed by pre-existing coding frameworks[34, 35, 47] and part inductively in response to the data.

**Table 1:** Codebook.

Tweet variable	Code*	Definition
Sentiment	Positive	Tweet is clearly in favour of standardised packaging – Tweet reports third party activity/position/opinion which has a positive spin – Tweet is understood to be positive in context of the Twitter conversation
	Negative	– Tweet is clearly opposed standardised packaging – Tweet reports third party activity/position/opinion which has a negative spin – Tweet is understood to be negative in context of the Twitter conversation
	Neutral	– Tweet only states facts about standardised packaging with no inflection at all
	Unclear	– Tone of tweet towards standardised packaging is unclear with no implication of either a positive or negative message.
Theme	Health benefits	– Standardised packaging will benefit health – Packaging is important to marketing – There is evidence to support standardised packaging – Standardised packaging will reduce tobacco sales – Evidence shows Australian standardised packaging works
	Non-health reasons to enact policy	– Standardised packaging will reduce tobacco company profits – Standardised packaging has public support – Standardised packaging will spread to other countries – Standardised packaging will not cost jobs – Standardised packaging will not increase the illicit trade in tobacco – Standardised packaging will not contravene intellectual property laws or trade agreements – The Government should do more for public health
	No health benefits	– Standardised packaging will not benefit health – Packaging is not important to marketing – There is no evidence to support standardised packaging – Standardised packaging will not reduce tobacco sales – Australian standardised packaging did not work
	Non-health reasons to reject policy	– Standardised packaging will cost jobs, – Standardised packaging will increase the illicit trade in tobacco and – Standardised packaging will contravene intellectual property laws and trade agreements – Standardised packaging for tobacco will spread to other products (slippery slope) – Standardised packaging will marginalise smokers and tobacco companies Government should not interfere with business
	No Theme	– Tweet contains no specific comments on the effect of standardised packaging
	Unclear	– Meaning of Tweet text is unclear
Purpose	Informative	– Providing information – Selling something or promoting a product
	Argument	– Making an argument – Promoting a campaign
	Critical	– Criticising alternative points of view in an abusive, political or satirical way – Exposing perceived wrongdoing or malpractice
	Discursive	– Raising a point or question for discussion
	Unclear	– Purpose is unclear
User	Health sector	– Twitter user is recognised or self-identifies as being a health professional, academic or representing a not-for-profit organisation (excludes government)
	Tobacco industry-linked	– Twitter user is recognised or self-identifies as being linked to the tobacco industry (includes company employees and industry-funded front groups and think tanks)
	Neither	– Twitter user appears to be neither health sector nor linked to the tobacco industry
Location	Australia	– Twitter user identified themselves as being located in Australia
	UK	– Twitter user identified themselves as being located in the UK
	US	– Twitter user identified themselves as being located in the US
	Rest of the world	– Twitter user identifies themselves as being located in another part of the world including Canada, Ireland, New Zealand, Philippines as well as Africa, Asia, Caribbean, Middle East and South America
	No data	– Twitter user provided no location information
Evidence mentioned	Yes	– Specific evidence or the concept of evidence is mentioned in the tweet
	No	– The concept of evidence does not occur at all in the tweet
URL linked?	Yes	– A working URL was included in the tweet.
	No	– No working website URL was included in the tweet.
Quality of evidence cited in URL	Cites peer-reviewed journal article(s)	– URL includes references to peer-reviewed journal articles relating to standardised tobacco packaging
	Refers to non-peer-reviewed research or evidence	– URL includes references to other specific examples of research e.g. academic books, government, charity or private company reports or to unspecified research relating to standardised tobacco packaging
	Does not refer to research or evidence	– URL does not include any references to evidence or research relating to standardised packaging
URL Author	Health sector	– URL author is recognised or self-identifies as being a health professional, academic or representing not-for-profit organisation (excludes government)
	Tobacco industry-linked	– URL author is recognised or self-identifies as being linked to the tobacco industry (includes company employees and industry-funded front groups and think tanks)
	Neither	– URL author appears to be neither health sector nor linked to the tobacco industry

*Coding categories were based on those developed and used by Evans-Reeves *et al*., Hatchard *et al*. and Love *et al*. [34, 35, 47].

### Inter-coder reliability

To test inter-coder reliability, 20% of included Tweets were second-coded. Mean relevance inter-coder reliability across Times 1 and 2 was 96.25% with a Krippendorff's alpha coefficient of 0.875. For fine coding, agreement ranged from 83.2% to 98% and all variables fell above the recommended 0.8 score for reliability[48].

### Data analysis

Using SPSS, Chi-Squared analyses were conducted to examine relationships between time and all Tweet characteristics. Standardised residuals were examined to explore the relative significance of the categories within variables: values lying outside +1.96 are significant at p<0.05, outside ±2.58 are significant at p<0.01, and outside ±3.29 are significant at p<0.001 [49].

## Results

### Tweet volume, sentiment, theme and purpose

In our sample, 49% (508/1038) of all Tweets were in favour of standardised packaging and 19% (201/1038) were opposed. There were significant differences in the sentiment (p<0.001), theme (p<0.001) and purpose (p<0.001) of Tweets between Times 1 and 2 (Table 2), with Time 2 characterised by a greater proportion of negative and critical Tweets and by fewer Tweets annunciating specific arguments supporting standardised packaging than Time 1 (Table 2).

**Table 2:** Changes in Tweet and Twitter user characteristics between Times 1 and 2, n = 1038.

Tweet variable	Code	Example of Tweet*	Time 1	Time 2	All	Standardised residuals (*z* scores)**	Overall significance
Sentiment	Positive	The Government supports tobacco standardised packaging: This is an important step for preventing children from smoking.	337	171	508	*z* **=** **±5.4**	**χ**^**2**^ **= 133.9, df = 3, p<0.001**
	Negative	Plain packaging for tobacco is illiberal. It will be a Smugglers' Charter and could cost taxpayers billions.	70	131	201	*z* **=** **±2.9**	
	Neutral	Government announce they will legislate on plain packaging for cigarettes before general election.	38	142	180	*z* **=** **±5.3**	
	Unclear	Why do UKIP oppose plain packaging for tobacco products? It would give them more space to write their policies.	68	81	149	*z* = ±0.7	
Theme	Health benefits	Plain packaging has potential to save lives; the Government is progressing it to support the next generation’s health.	157	83	240	***z* =** **±3.5**	**χ**^**2**^ **= 201.2, df = 5, p<0.001**
	Non-health reasons to enact policy	Research shows Australian smokers now support plain packaging.	136	11	147	***z* =** **±7.3**	
	No health benefits	More fake 'evidence' for 'success' of #plainpacks which makes no mention of children. #ConTrick	31	45	76	*z* = ±1.1	
	Non-health reasons to reject policy	More common sense on @bbcquestiontime: Plain packaging on tobacco WILL make counterfeiting easier.	35	61	96	*z* = ±1.8	
	No Theme	New Zealand progresses towards plain packaging for tobacco products.	148	319	467	***z* =** **±5.4**	
	Unclear	Photo: plain tobacco packaging	6	6	12	*z* = ±0.0	
Purpose	Informative	Australia is the only state that has plain packaging for cigarettes.	316	240	556	*z* **=** **±2.5**	**χ**^**2**^ **= 47.5, df = 4, p<0.001**
	Argument	Plain packaging is a logical step for Canada to reduce tobacco marketing and smoking and save lives.	85	120	205	*z* = ±1.6	
	Critical	Plain packaging on cig packs will give politicians more room to plan their policies.	49	117	166	*z* **=** **±3.6**	
	Discursive	Is there an advantage for a tobacco brand to package its product in plain packaging first?	61	44	105	*z* = ±1.2	
	Unclear	Govt.: "We're introducing plain packs for tobacco [2 days later] "Ha ha, You believed us!" *tweets pics of diseased lungs*	2	4	6	*z* = ±0.6	
Twitter User Sector	Health sector	Australian smokers like plain packaging rules.	90	40	130	*z* = **±3.2**	**χ**^**2**^ **= 23.9, df = 2, p<0.001**
	Tobacco industry-linked	Plain packaging will be pointless. Let's thank smokers for funding so much through tax. #bbcqt	15	13	28	*z* = ±0.3	
	No apparent links to health or tobacco industry	Positive: #philipmorris complaining in #Economist that plain packs aim to 'disparage' their products. No, they aim to stop you killing people Negative: Making smokers buy their cigarettes in plain packs will not save the NHS or them. #bbcqt	408	472	880	*z* = ±1.3	
Twitter User Location	Australia	Aussie smokers happy with plain packaging shows recent survey @guardian	85	27	112	*z* **=** **±3.9**	**χ**^**2**^ **= 67.5, df = 4, p<0.001**
	UK	Public health advocates are pushing soda taxes and plain packaging	131	215	346	*z* **=** **±3.0**	
	US	John Oliver on big tobacco; applauding Australia’s plain packaging laws. #JeffWeCan	51	30	81	*z* = ±1.7	
	Rest of the world	British government vote to require tobacco firms to sell cigarettes in plain packaging. [Tweet from Singapore]	76	45	121	*z* **=** **±2.1**	
	No data	n/a	170	208	378	*z* = ±1.2	

* Tweets paraphrased to protect anonymity of users, in line with British Psychological Society Ethics Guidelines for Internet-mediated Research 2014

** Categories which significantly contribute to the overall chi squared statistic have *z* scores outside ±1.96 (significant at p<0.05), outside ±2.58 (significant at p<0.01), and outside ±3.29 (significant at p<0.001). All significant scores are highlighted in bold.

At Time 1, nearly two thirds of Tweets (66%, 337/513) expressed a positive *sentiment* towards standardised packaging. In contrast, at Time 2, the proportion of positive Tweets halved compared to Time 1 (33%, 171/525) and Tweets expressing a negative sentiment towards standardised packaging increased from 14% (70/513) at Time 1 to 25% (131/525) at Time 2. Neutral Tweets were also more prevalent at Time 2, rising from 14% (70/513) to 25% (131/525). With respective *z* scores of ±5.4, ±5.3 and +2.9 (Table 2), the change in the proportion of positive, neutral and negative Tweets between Times 1 and 2 were found to significantly contribute to the overall chi squared statistic.

Results for *theme* partially reflect those of sentiment (Table 2). The decline in the proportion of positive Tweets in the sample at Time 2 is mirrored by a significant decline in both Tweets detailing specific pro-standardised packaging arguments relating to health benefits (157/513 at Time 1, 83/525 at Time 2, *z* = ±3.5) and those describing additional reasons to enact the policy such as public support and the negative effect on tobacco industry profits (136/513 at Time 1, 11/525 at Time 2, *z* = ±7.3). However, no significant increase in the proportion of Tweets rejecting health benefits and highlighting other reasons not to enact standardised packaging, such as a rise in illicit trade or contravening intellectual property laws and trade agreements was observed at Time 2. Instead, Time 2 was characterised by a significantly greater proportion of Tweets with no specific theme (148/513 at Time 1, 319/525 at Time 2, *z* = ±5.4).

In terms of *purpose*, Time 2 showed a significant increase in the proportion of critical Tweets (characterised by abusive, political or satirical criticism and/or accusations of malpractice or misrepresentation) from 10% (49/513) of the sample at Time 1 to 22% (117/525) at Time 2 (*z* = ±3.6). Two thirds of critical Tweets at Time 2 were political and tended to refer to the imminent general election. Time 2 also showed a significantly lower proportion of Tweets with an informative purpose (*z* = ±2.5). For the most part, informative Tweets were presenting facts about standardised packaging of tobacco products policy, implementation and effects. Only three of these were marketing Tweets.

### Twitter-user characteristics

A majority of Tweets in the sampled data were published by independent Twitter-users with no discernible links to either the health sector or the tobacco industry (85%, 880/1038) (Table 2). However, there were significant differences in the profile of Twitter-users between Times 1 and 2 (Table 2). Mirroring the reduction in the proportion of Tweets which were positive about standardized packaging, Tweets were more likely to be from users linked to the health sector at Time 1 (18%, 90/513) than at Time 2 (8%, 40/525, *z* = ±3.2).

Location information was provided by users for 660 Tweets in our sample (Table 2). Of these, Tweets originated from all over the world, but more than half (52%, 346/660) were from the UK. A greater proportion of Tweets originated from Australia at Time 1 than at Time 2 (*z* = ±3.9): Time 1 included the publication of a research paper by Swift *et al*. which found increased support for the policy among Australian smokers for the policy following implementation[50]. At Time 2, following the UK Government announcement, a significantly higher proportion were from the UK (*z* = ±3.0). Tweets from the rest of the world–including Africa, Asia, other European countries, the Middle East, Canada, the Caribbean, New Zealand and the Philippines–saw a relative decline at Time 2 (*z* = ±2.1).

### Sharing of evidence and research via Tweets

Evidence and research were shared in 58% (605/1038) of sampled Tweets in either the text of the Tweet itself and/or in the 258 unique URL-linked webpages and images. One in 10 (105/1038) Tweets *both* mentioned evidence *and* linked to a URL; 45% (465/1038) solely included a URL which mentioned evidence or research; 3% (35/1038) only mentioned evidence or research in the Tweet itself.

The volume, quality and source of evidence and research mentioned and shared via Twitter differed significantly between Tweets sampled at Times 1 and 2 (p<0.001, Table 3). Time 1 Tweets were more likely to mention evidence (*z* = ±3.8), to share URLs citing peer-reviewed research (*z* = ±6.7), and to share URLs originating from the health sector (*z* = ±5.5). At Time 2, Tweets were more likely to share URLs which referred to non-peer-reviewed research or evidence (*z* = ±5.6) or to no evidence at all (*z* = ±2.5), and to include URLs originating from neither the health nor tobacco sectors (*z* = ±2.7).

**Table 3:** Relationship between time and evidence dissemination.

Tweet variable	Code	Time 1	Time 2	All	Standardised residuals (*z* scores)*	Overall significance
Evidence mentioned, n = 1038	Yes	101	39	140	*z* **=** **±3.8**	**χ**^**2**^ **= 33.4, df = 1, p<0.001**
	No	412	486	898	*z* **=** ±1.5	
URL linked, n = 1038	Yes	373	353	726	*z* = ±0.7	χ^2^ = 5.1, df = 2, p = 0.078
	No	128	150	278	*z* = ±0.8	
	No document access	12	22	34	*z* = ±1.2	
Quality of evidence cited in URL, n = 726	Cites peer-reviewed journal article(s)	250	70	320	*z* = **±6.7**	**χ**^**2**^ **= 168.7, df = 2, p<0.001**
	Refers to non-peer-reviewed research or evidence	65	185	250	*z* = **±5.6**	
	Does not refer to research or evidence	58	98	156	*z* = **±2.5**	
URL author sector, n = 726	Health sector	110	18	128	*z* = **±5.5**	**χ**^**2**^ **= 76.5, df = 2, p<0.001**
	Tobacco industry-linked	27	23	50	*z* = ±0.3	
	Neither	236	312	548	*z* = **±2.7**	

* Categories which significantly contribute to the overall chi squared statistic have *z* scores outside ±1.96 (significant at p<0.05), outside ±2.58 (significant at p<0.01), and outside ±3.29 (significant at p<0.001). All significant scores are highlighted in bold.

Significant differences were also observed in the quality of the research cited by different Twitter-users in the sample (p<0.001, Table 4). Overall, URLs citing peer-reviewed journal research were more likely to be written by health authors (*z* = ±4.6) and more likely to be Tweeted by health sector Twitter-users (*z* = ±2.7). Although, this was more common at Time 1 than at Time 2. Positive Tweets were also significantly more likely to include material citing peer-reviewed research (z = +7.3).

**Table 4:** Relationship between Sentiment, Twitter user and URL author sector and evidence quality, n = 726.

Tweet variable	Code	Cites peer-reviewed research	Does not cite peer-reviewed research	All	Standardised residuals (*z* scores)*	Overall significance
URL author sector	Health sector	**95**	**33**	128	*z* **=** **±4.6**	**χ**^**2**^ **= 67.6, df = 2, p<0.001**
	Tobacco industry-linked	**8**	**42**	50	*z* **=** **±2.7**	
	Neither	217	331	548	*z* = ±1.4	
Twitter user sector	Health sector	**71**	**45**	116	*z* **=** **±2.5**	**χ**^**2**^ **= 22.6, df = 2, p<0.001**
	Tobacco industry-linked	**3**	18	21	*z* **=** ±1.8	
	Neither	246	343	589	*z* **=** ±0.8	
Sentiment	Positive	**286**	**116**	402	*z* **=** **±7.3**	**χ**^**2**^ **= 272.2, df = 3, p<0.001**
	Negative	**13**	**98**	111	*z* **=** **±4.6**	
	Neutral	**8**	**143**	151	*z* **=** **±6.4**	
	Unclear	13	49	62	*z* **=** **±2.4**	

* Categories which significantly contribute to the overall chi squared statistic have *z* scores outside ±1.96 (significant at p<0.05), outside ±2.58 (significant at p<0.01), and outside ±3.29 (significant at p<0.001). All significant scores are highlighted in bold.

## Discussion

This study shows that, following the UK Government’s announcement of a parliamentary vote on standardised tobacco packaging in January 2015, Twitter communication about the policy measure changed. Prior to the announcement, Tweets which expressed a positive sentiment towards the policy comprised approximately two thirds of Tweets. In the wake of the announcement, the proportion of sampled Tweets that were negative towards standardised packaging increased (from one in ten to one in five), while the proportion of positive Tweets dropped to a third. At Time 2, Tweets from health sector users and those sharing peer-reviewed health research were also relatively less visible in our sample. As the total volume of Tweets was nearly three times greater at Time 2 than at Time 1, it is likely that the absolute volume of positive Tweets remained relatively stable across the two time periods; but that negative Tweets significantly increased in volume. Few tobacco industry-linked Tweets were identified in the sample with no significant change observed after the Government’s announcement. There was little evidence in the sampled data of social bot activity.

These findings suggest that the health community used Twitter proactively as a tool for dissemination of policy-related research: new peer-reviewed research was published supporting standardised packaging at both Times 1 and 2[50–54]. Indeed, we know that the Plain Packs Protect Partnership had a Twitter presence in 2012–14: @PlainPacks which it used to campaign in favour of standardised packaging. However, this supportive Twitter activity is likely to have been relatively less visible at Time 2 as the salience of the issue on this social media platform increased. The results further suggest that those opposed to standardised packaging were using Twitter in a more reactive way than were their health counterparts, conceiving it mainly as a venue for protest, in line with that of the tobacco industry and tobacco retailers’ opposition at Time 2 [41], rather than for evidence communication. However, the lack of evidence communication is also likely to reflect that there was no independent, high-quality research that supported opposition arguments to standardised packaging. Indeed the evidence against standardised packaging has predominantly come from a narrow base of industry-related sources and is not peer-reviewed. Its low quality was remarked upon in the UK High Court ruling on standardised packaging in 2016 which confirmed that the policy was lawful.[19, 33–35, 55, 56].

The findings provide insight regarding three aspects of existing knowledge on the use of Twitter in health policy conflicts. First, previous research has found that Twitter messages validly reflect the political landscape (even being used to predict election results)[5]. Although parliamentary voting is somewhat different from public elections, the sentiment analysis of this dataset does suggest a large body of public support for the policy. However, taken separately, the data at Time 2 did not wholly reflect the parliamentary vote in favour of standardised packaging in the UK in March 2015. Instead, Twitter provided a venue for the expression of UK-based negative reaction to the Government’s announcement.

Second, existing research has pointed to the importance of social media to both non-profitmaking organisations and corporations[57–59]. The present research shows how public health academics and advocates are using Twitter to share and promote peer-reviewed evidence on public health policy options. They are doing this by providing bite-sized summaries of new research in tweets and by sharing URLs of full academic peer-reviewed research articles, of plain English blogs written by academics themselves, and of media reports of research. In doing so, our research adds more weight to calls for public health advocates to make effective use of Twitter and other social media tools to support campaigns for policy change.[60, 61] A key route for achieving this is for academic research to be translated into accessible brief formats suitable for public communication of science, either by academics themselves or in collaboration with advocacy groups.[62–64]

Third, the findings challenge the prevailing view of Twitter as being a primary cite for automated activity, particularly in relation to marketing. Unlike several research studies examining e-cigarette-related content on Twitter[65–67] and contemporary debates about the role of social media in ‘fake news’, the relative absence of marketing Tweets and social bots [45, 46] in the present dataset is surprising. The only two examples to be found in the data sought to promote cigarette case use as a means of circumventing standardised packaging legislation. This finding, which does not chime with other research from the field, may be due to this study’s search terms, which focused on a public health policy, rather than a product, brand or company. As such, the study provides scant evidence that opponents of standardised packaging were using automated accounts to exploit Twitter’s potential to influence, and distort perceptions of, wider public opinion or that marketeers were exploiting this policy issue to sell tobacco-related products.

In terms of strengths, this study has opened up a new avenue of investigation of the use of Twitter in health policy conflicts and provides insights into the different ways in which health policy advocates and opponents may be using this social media platform to promote their policy position. The inclusion of re-Tweets and of multiple Tweets by the same users meant our dataset particularly reflected the level of those Twitter users’ engagement with the issue of standardised packaging. However, the low frequency of Tweets which could clearly be linked to the tobacco industry in this dataset precluded specific analysis of tobacco industry-linked Twitter activity. Future work could seize the opportunity for additional analysis of Twitter handles, hashtags and arguments used by the tobacco industry at present. This would helpfully supplement existing analyses of tobacco industry arguments which have drawn mainly on public consultation data and advertisements.[19, 33–35, 68] This deficit could be addressed in future studies by comparatively analysing pre-identified industry-linked Twitter profiles and content, using a method similar to that of Kavuluru & Sabbir’s (2016) work on e-cigarettes[69]. This approach could also add to existing literature [19, 34] by unearthing previously hidden relationships between tobacco companies and supposedly independent third-parties and could also be extended to other health-harming industries, such as alcohol and sugar-sweetened beverage producers and retailers.

To conclude, this study shows that Twitter can be used to examine public sentiment on public health policy and reactions to policy events. Microblogging sites such as Twitter can reflect offline policy debates and can be a particularly useful tool for sharing public health research and advocacy messages. (60, 61) The research highlights in particular the need for public health advocates to prepare for backlashes at key events and times during policy debates and to bolster their social media strategy accordingly. For example by increasing Tweet volume and communicating both supportive evidence and evidence-based counter-arguments to industry claims regarding “negative unintended consequences” of policies. Microblogging platforms like Twitter present an opportunity for disseminating and promoting lay summaries of public health research particularly at key policy moments–an opportunity which can be taken up more frequently by public health academics and advocates together both within countries and internationally.
